# Efficacy and safety of dulaglutide in patients with type 2 diabetes: a meta-analysis and systematic review

**DOI:** 10.1038/srep18904

**Published:** 2016-01-08

**Authors:** Lin Zhang, Mei Zhang, Yuwei Zhang, Nanwei Tong

**Affiliations:** 1 Department of Endocrinology and metabolism, West China Hospital, Sichuan University; The third affiliated hospital of Chengdu University of Traditional Chinese Medicine; 2 Department of Laboratory Medicine, West China Hospital, Sichuan University; 3 Department of Endocrinology and metabolism, West China Hospital, Sichuan University, Chengdu 610041, Sichuan Province, P.R. China

## Abstract

A meta-analysis was conducted to assess the clinical efficacy and safety of dulaglutide in patients with type 2 diabetes mellitus (T2DM). Medline, Embase, Cochrane Library and www. clinicaltrials. gov (up to February 15^th^, 2015) were searched. Randomized controlled trials comparing dulaglutide to other drugs for T2DM were collected. Twelve RCTs were included, and the overall bias was low. As the monotherapy, compared with control (placebo, metformin and liraglutide), dulaglutide resulted in a significant reduction in HbA1c (WMD, −0.68%; 95% CI, −0.95 to −0.40), FPG (WMD, −0.90 mmol/L; 95% CI, −1.28 to −0.52), a similar risk of hypoglycemia (7.8% vs. 10.6%), less body weight loss (WMD, 0.51 kg; 95% CI, 0.27 to 0.75). As an add-on intervention with oral antihyperglycemic medication (OAM) and insulin, compared with control (placebo, sitagliptin, exenatide, liraglutide and glargine), dulaglutide lowered HbA1c (WMD, −0.51%; 95% CI, −0.68 to −0.35) and body weight significantly (WMD, −1.30 kg, 95% CI, −1.85 to −1.02) notably, and elicited a similar reduction in FPG (WMD, −0.19 mmol/L; 95% CI, −1.20 to 0.82), an similar incidence of hypoglycemia (24.5% vs. 24.5%). This meta-analysis revealed the use of dulaglutide as a monotherapy or an add-on to OAM and lispro appeared to be effective and safe for adults with T2DM.

Glucagon-like peptide-1 (GLP-1) is released from neuroendocrine intestinal L-cells, and can reduce glucose levels by promoting the secretion of insulin and decreasing glucagon, delaying gastric emptying, and reducing food intake^1^,^2^. However, endogenous GLP-1 is easily degraded and inactivated by the protease dipeptidyl peptidase-4 (DPP-4), which cleaves the N-terminal histidine and alanine residues from GLP-1 (7–36) to generate GLP-1 (9–36) amide. This leads to a very short half-life of native GLP-1 (~1–2 min)^3^, and limits its clinical applications. The short half-life of GLP-1 has prompted efforts to identify novel agents to meet the clinical demands. Therefore, GLP-1 receptor agonists have been researched and developed.

GLP-1 receptor agonists can be divided into long-acting agents (liraglutide, dulaglutide, albiglutide, and exenatide long-acting release), which predominantly reduce fasting plasma glucose (FPG) levels, and short-acting agents (exenatide, lixisenatide), which notably lowers postprandial glucose (PPG) levels^4^. Dulaglutide (LY2189265; Eli Lilly and Company, USA) is a novel, long-acting GLP-1 receptor agonist that is administered via subcutaneous injection for the treatment of type 2 diabetes mellitus (T2DM), and has been approved by the United States Food and Drug Administration (FDA). Dulaglutide consists of two modified GLP-1 peptides that contain amino acid substitutions that protect it from inactivation by DPP-4 that are linked by small peptides to a modified human IgG4-Fc heavy chain to reduce immunogenicity and cytotoxicity and increase stability^5^. Dulaglutide is a large sized molecule (molecular weight. 59.7 kDa), which limits its renal clearance. This results in a half-life of ~4 days and a time-to-peak concentration of ~70 h; this allows for once-weekly dosing, which might improve patient compliance significantly^6^,^7^. Dulaglutide has been evaluated and is currently being evaluated in large-scale, long-term randomized trials specifically designed for the treatment of T2DM.

In this study we conducted a systematic review and meta-analysis to present an overview of the efficacy and safety of dulaglutide in subjects with T2DM.

## Results

### Search results

**Figure 1:**
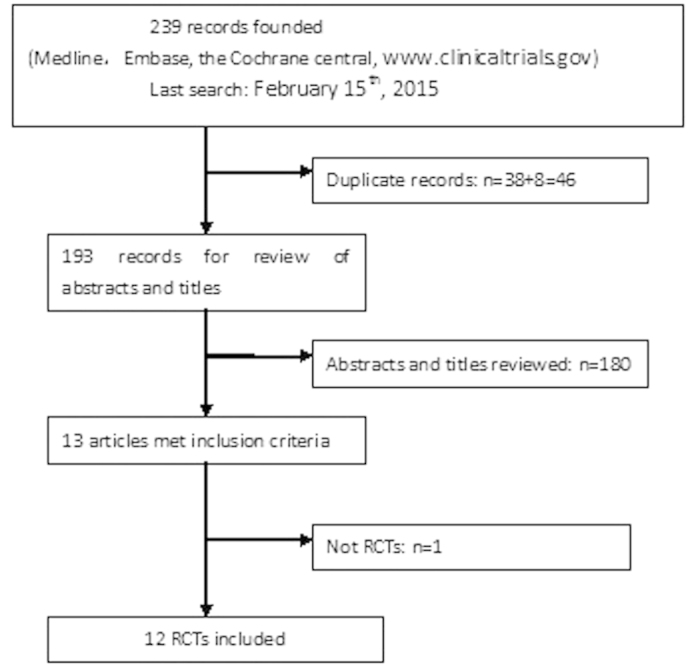
Flow diagram of study selection process.

The selection flow diagram is presented in Fig. 1. A total of 239 records were identified using the search strategies to screen the databases. Of these articles, 30 were duplicate records. After classifying the documents according to titles, abstracts, and full texts, twelve records^8^,^9^,^10^,^11^,^12^,^13^,^14^,^15^,^16^,^17^,^18^,^19^ were eligible for inclusion.

### Clinical characteristics

Details regarding patient demographics, baseline characteristics, and study arms are shown in Table 1. All of these RCTs provided information regarding the efficacy and safety of dulaglutide that was administered once weekly by subcutaneous injection in subjects with type 2 diabetes. The study durations were 12 to 104 weeks. The included trials were all multicenter, and were conducted in the United States, Puerto Rico, Canada, France, Japan, and other countries or areas; one study did not report the location^9^. A total of 4640 and 2801 patients with type 2 diabetes were in the dulaglutide group and control group respectively.

The doses of dulaglutide were fixed in eleven trials^9^,^10^,^11^,^12^,^13^,^14^,^15^,^16^,^17^,^18^,^19^, whereas the other study administered titrated and non-titrated doses of LY2189265 (LY)^8^: LY 0.5/1.0 (LY 0.5 mg for 4 weeks then 1.0 mg for 12 weeks), LY 1.0/1.0 (LY 1.0 mg for 16 weeks), and LY 1.0/2.0 (LY 1.0 mg for 4 weeks then 2.0 mg for 12 weeks). Placebo was used as the control group in six studies^8^,^9^,^11^,^13^,^15^,^19^, two used sitagliptin^10^,^11^, one used exenatide^13^, two used liraglutide^14^,^19^, and one used metformin^12^, three used glargine^16^,^17^,^18^. One study^12^ used dulaglutide as a monotherapy, compared with metformin and two trials^9^,^15^ compared dulaglutide monotherapy with placebo, one study compared with liraglutide^19^. In the add-on trials, metformin, sulfonylurea, thiazolidinedione, or other oral antihyperglycemic medication (OAM) and lispro were used as the background therapy in eight trials^8^,^10^,^11^,^13^,^14^,^16^,^17^,^18^.

In one study^11^ that lasted for 52 weeks, patients were randomized into dulaglutide (0.75 mg, 1.5 mg), sitagliptin, and placebo groups; the placebo group were switched to sitagliptin after 26 weeks. Guerci *et al.*^10^ reported some of the results of this study in an abstract. In another study^13^ that also lasted for 52 weeks, subjects were randomized into one of four groups: dulaglutide (0.75 mg, 1.5 mg), exenatide, and placebo. After 26 weeks the placebo-treated patients were switched to dulaglutide 1.5 mg or dulaglutide 0.75 mg.

### Methodological quality

**Figure 2:**
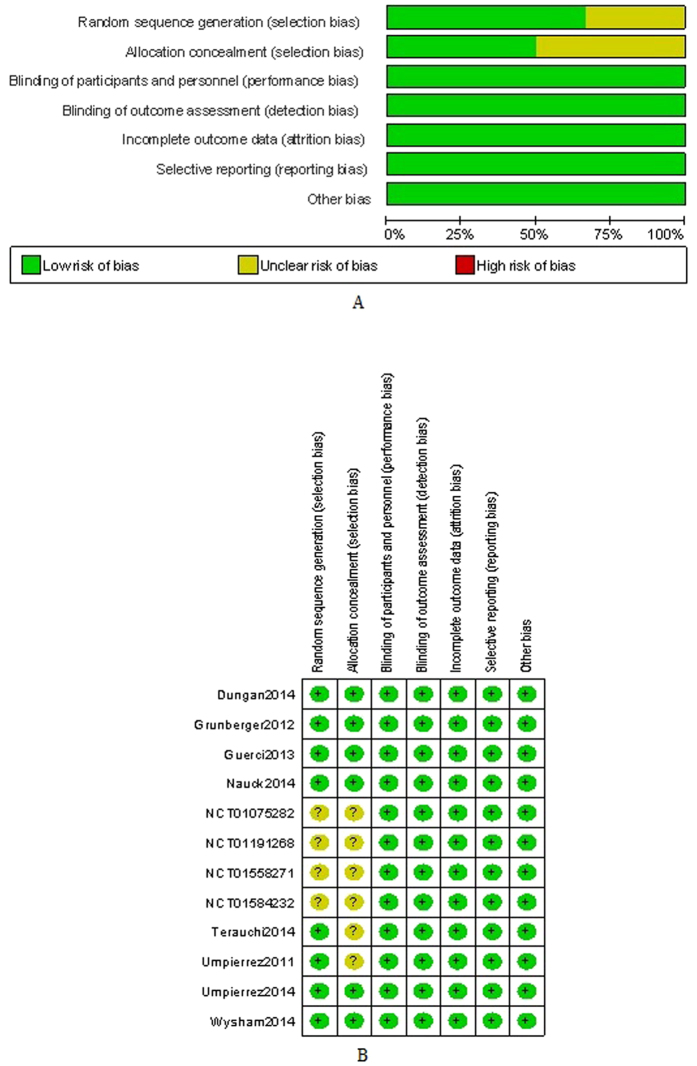
(**A**) Risk of bias graph (**B**) Risk of bias summary.

The risk of bias was estimated according to the Cochrane Collaboration’s risk of bias tool; the data were shown in Fig. 2(A,B). Random sequence generation was clear in eight RCTs^8^,^9^,^10^,^11^,^12^,^13^,^14^,^15^ but can not be obtained in the other four^16^,^17^,^18^,^19^. Similarly, allocation concealment was described explicitly in four RCTs but not in the other six^8^,^15^,^16^,^17^,^18^,^19^. The overall risk of bias in the included studies was low. Eli Lilly and Company funded all the trials. Intent-to-treat analysis was used to evaluate all randomized subjects who accepted at least one dose of the study treatment.

### HbA1c

**Figure 3:**
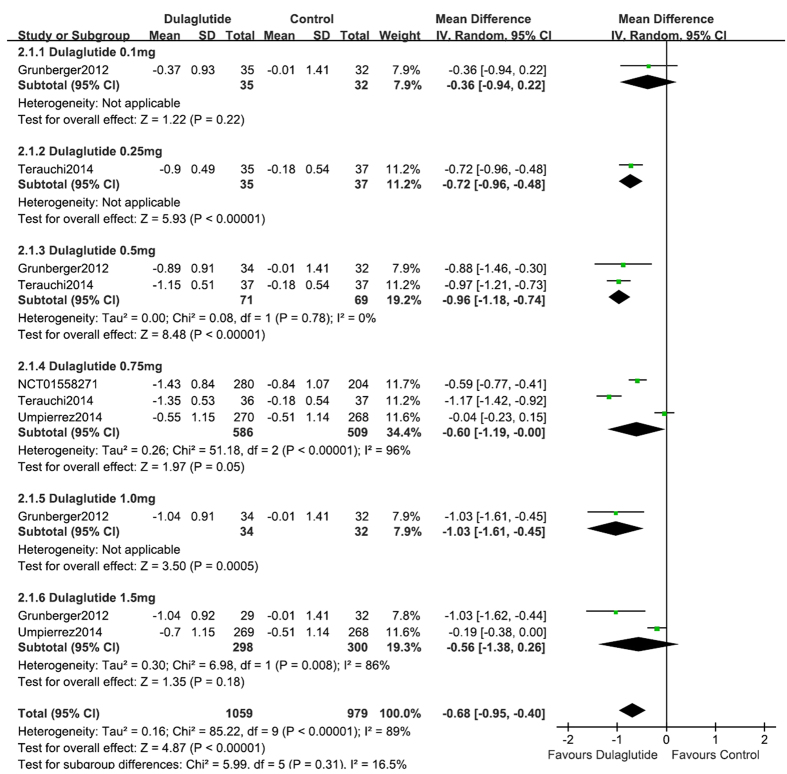
HbA1c: dulaglutide monotherapy vs. control.

As a monotherapy, dulaglutide (*n* = 1059) lowered HbA1c significantly compared with control (placebo, metformin and liraglutide) (*n* = 544)^9^,^12^,^15^,^19^ (WMD, −0.68%; 95% CI, −0.95 to −0.40) (Fig. 3). The percentage of patients that achieved HbA1c < 7% was significantly higher with dulaglutide (62.3%) than with control (44.9%) (RR, 2.24; 95% CI, 1.65 to 3.03). In addition, 40.5% of dulaglutide-treated subjects achieved HbA1c ≤6.5%, compared with 27.0% of those in the control groups (RR, 2.26; 95% CI, 1.56 to 3.28).

In the three trials^9^,^15^,^19^ that compared dulaglutide monotherapy with placebo, dulaglutide reduced HbA1c significantly (WMD, −1.00%; 95% CI, −1.27 to −0.73). Compared with placebo, dulaglutide monotherapy achieved a greater proportion of patients with HbA1c <7% (68.1% vs. 10.9%, respectively; RR, 4.97; 95% CI, 3.66 to 6.73) and ≤6.5% (42.1% vs. 2.9%; RR, 10.52; 95% CI, 5.66 to 19.54). When dulaglutide (0.75 mg, 1.5 mg) was used as a monotherapy and compared with metformin (titrated up to 2000 mg/day or at least 1500 mg/day depending upon tolerability), dulaglutide, and metformin reduced HbA1c to a similar extent (WMD, −0.11%; 95% CI, −0.25 to 0.02). The percentage of patients that achieved HbA1c <7% (56.8% vs. 50.0%; RR, 1.16; 95% CI, 1.05 to 1.29) and ≤6.5% (39.0% vs. 28%; RR, 1.67; 95% CI, 1.09 to 2.56) was higher with dulaglutide compared with metformin, respectively^12^. Compared with liraglutide (0.9mg), dulaglutide (0.75 mg) once weekly led to the similar reduction of HbA1c (−1.43% vs. −1.33%; WMD, −0.10%; 95% CI, −0.27 to 0.07), similar percentage of patients that achieved HbA1c <7% (71.4% vs. 69.1%; RR, 1.12; 95% CI, 0.71 to 1.75) and ≤6.5% (50.0% vs. 49.3%; RR, 1.03; 95% CI, 0.68 to 1.55)^19^.

**Figure 4:**
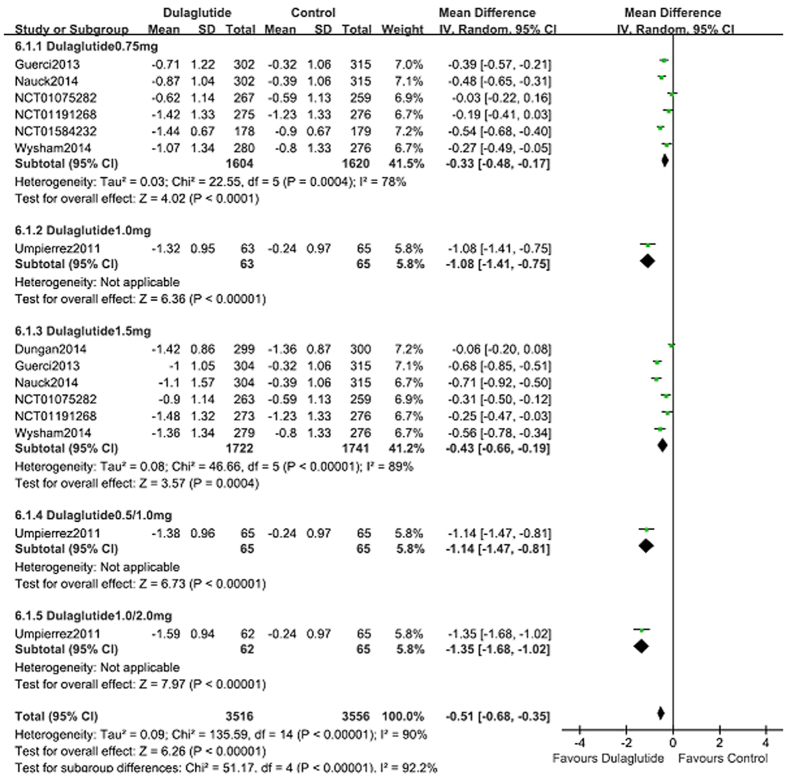
HbA1c: dulaglutide add-on to active drugs vs. control.

When used as an add-on to OAM and lispro, compared with control (placebo, sitagliptin, exenatide, liraglutide and glargine; *n* = 2328), dulaglutide (*n* = 3581) lowered HbA1c notably (WMD, −0.51%; 95% CI, −0.68 to −0.35; Fig. 4)^8^,^10^,^11^,^13^,^14^,^16^,^17^,^18^. More patients reached the HbA1c target of <7.0% with dulaglutide (55.6%) than with control (43.6%) (RR, 1.36; 95% CI, 1.21 to 1.53). The percentage of patients reached the HbA1c target of ≤6.5% in the dulaglutide was more than in control groups (36.9% vs. 32.1%; RR, 1.47; 95% CI, 1.19 to 1.81).

As an add-on to OAM, dulaglutide lowered HbA1c notably compared with placebo; the pooled HbA1c WMD was −0.78% (95% CI, −1.04 to −0.51)^8^,^11^,^13^. The proportion of patients that reached HbA1c <7% (57.4% vs. 44.7%; RR, 1.40; 95% CI, 1.18 to 1.67) and ≤6.5% (35.5% vs. 19.0%; RR, 1.84; 95% CI, 1.28 to 2.65) was markedly greater in the dulaglutide group than in the placebo control group. As an add-on to metformin, dulaglutide (0.75 mg and 1.5 mg; *n* = 1212) lowered HbA1c significantly compared with sitagliptin (100 mg qd; *n* = 630)^10^,^11^ (WMD, −0.56%; 95% CI, −0.71 to −0.41). Dulaglutide increased the proportion of patients that reached HbA1c target of <7% (51.4% vs. 32.1%; RR, 1.60; 95% CI, 1.45 to 1.77) and ≤6.5% (35.5% vs. 19.0%; RR, 1.86; 95% CI, 1.54 to 2.26) compared with the sitagliptin group. Both of the dulaglutide groups were superior to sitagliptin.

When compared with glargine, as an add-on to OAM and lispro, dulaglutide lowered HbA1c notably (WMD: −0.27% ; 95% CI, −0.46 to −0.08)^16^,^17^,^18^. The proportion of patients that reached HbA1c <7% (52.6% vs. 41.6%; RR, 1.30; 95% CI, 1.12 to 1.51) and ≤6.5% (33.4% vs. 23.8%; RR, 1.63; 95% CI, 1.37 to 1.94) (data not shown) was markedly greater in the dulaglutide group than in the glargine control group^16^,^17^,^18^.

Wysham *et al.*^13^ performed a head-to-head comparison of dulaglutide (0.75 mg and 1.75 mg QW; *n* = 559) and another GLP-1 receptor agonist, exenatide (10 μg BID; n = 276) using metformin + pioglitazone as the background treatment. Data revealed that the ability to reduce HbA1c (WMD, −0.41%; 95% CI, −0.70 to −0.13) and the percentage of patients that attained HbA1c target of <7% (64.8% vs. 48.9%; RR, 1.32; 95% CI, 1.11 to 1.58) was significantly greater with dulaglutide. At 26 weeks the percentage of patients that achieved the HbA1c goal of ≤6.5% was 63%, 53%, 38%, and 24% in the dulaglutide 1.5 mg, dulaglutide 0.75 mg, exenatide, and placebo arms, respectively (*P* < 0.001 for all comparisons).

Dungan *et al.*^14^ compared dulaglutide (1.5 mg) once weekly with liraglutide (1.8 mg) once daily using metformin as the background treatment. The mean reduction in HbA1c was −1.42% and −1.36%, respectively; there was no significant difference between groups (WMD, −0.06%; 95% CI, −0.20 to 0.08). The proportion of patients that achieved HbA1c target <7.0% was 68% and 55%, and HbA1c target ≤6.5% was 55% and 51% in the dulaglutide and liraglutide groups respectively. There were no significant differences between the two groups.

### Blood glucose

**Figure 5:**
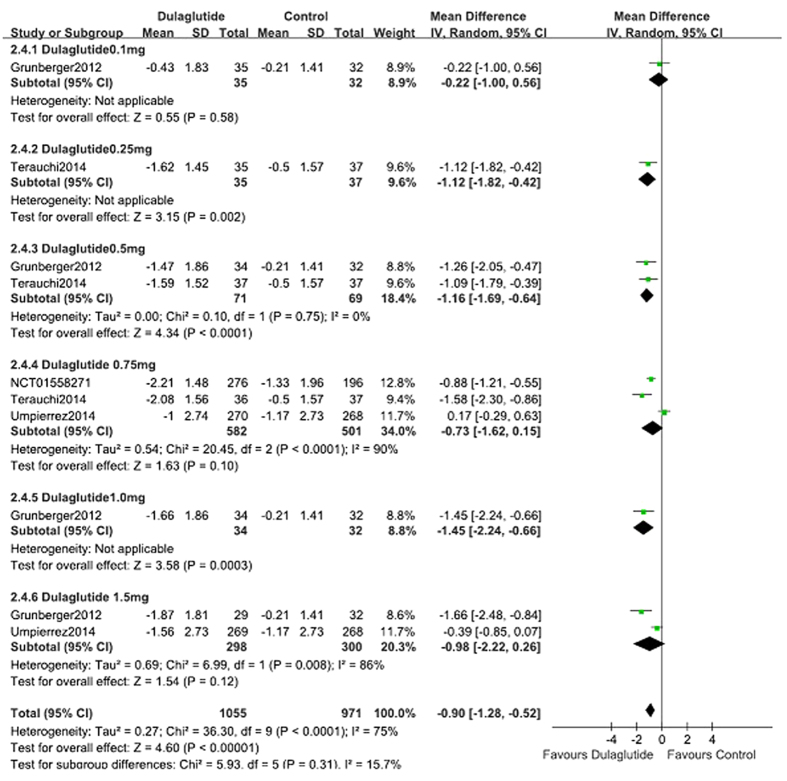
FPG: dulaglutide monotherapy vs. control.

When used as a monotherapy dulaglutide (*n* = 1059) lowered FPG significantly compared with control (placebo, metformin and liraglutide; *n* = 544; WMD, −0.90 mmol/L; 95% CI, −1.28 to −0.52) (Fig. 5)^9^,^12^,^15^,^19^. Compared with placebo, dulaglutide monotherapy also reduced FPG significantly (WMD, −1.74 mmol/L; 95% CI, −1.97 to −1.51)^9^,^15^,^19^. When compared with metformin monotherapy (the titration of up to 2000 mg/day or at least 1500 mg/day), 0.75 or 1.5 mg dulaglutide reduced FPG; however, there was no difference between groups (WMD, −0.11 mmol/L; 95% CI, −0.66 to 0.44). Compared with liraglutide, dulaglutide monotherapy resulted in similar reduction of FPG (WMD, 0.00 mmol/L; 95% CI, −0.31 to 0.31).

**Figure 6:**
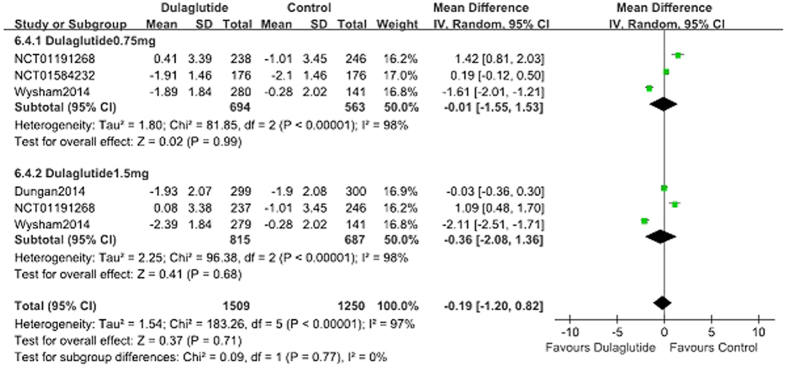
FPG: dulaglutide add-on to active drugs vs. control.

When used as an add-on therapy to OAM and lispro, dulaglutide (*n* = 1627) similarly reduced FPG levels and control (placebo, liraglutide and glargine; *n* = 917) (WMD, −0.19 mmol/L; 95% CI, −1.20 to 0.82)^13^,^14^,^16^,^17^,^18^ (Fig. 6). Compared with placebo, the changes in FPG were notably greater in the dulaglutide group (WMD, −1.86 mmol/L; 95% CI, −2.35 to −1.37)^13^. The reduction in FPG was similar in the dulaglutide (1.5 mg) and liraglutide (1.8 mg) groups (WMD, −0.03 mmol/L; 95% CI. −0.36 to 0.30)^14^, less reduction in dulaglutide group compared with glargine (WMD, 0.87 mmol/L; 95% CI, 0.04 to 1.69)^16^,^17^,^18^.

### Bodyweight

When administered as a monotherapy, dulaglutide (*n* = 1059) lowered bodyweight less than control (placebo, metformin and liraglutide; *n* = 544)^9^,^12^,^15^,^19^ (WMD, 0.51 kg; 95% CI, 0.27 to 0.75) (see Supplementary Fig. S1 online). Dulaglutide monotherapy reduced bodyweight less than placebo (WMD, 0.65 kg; 95% CI, 0.28 to 1.03; Figure S21)^9^,^15^, and exhibited a similar reduction in bodyweight as did metformin (WMD, 0.40 kg; 95% CI, −0.52 to 1.31)^12^ and liraglutide (WMD, 0.34 kg; 95% CI, −0.14 to 0.82)^19^.

As an add-on intervention with OAM and lispro, dulaglutide (*n* = 3581) lowered bodyweight significantly compared with control (placebo, sitagliptin, exenatide, liraglutide and glargine; *n* = 2328) (WMD, −1.30 kg, 95% CI, −1.85 to −1.02) (see Supplementary Fig. S2 online)^8^,^10^,^11^,^13^,^14^,^16^,^17^,^18^. As an add-on to OAM, dulaglutide lowered bodyweight notably compared with placebo (WMD, −1.71 kg; 95% CI, −2.36 to −1.07)^8^,^13^, and compared with 100 mg QD sitagliptin (WMD, −1.12 kg; 95% CI, −1.44 to −0.79)^10^,^11^. In addition, dulaglutide treatment resulted in a similar change in bodyweight as did exenatide (WMD, 0.48 kg; 95% CI, −1.04 to 1.99)^13^. Dulaglutide (1.5 mg) reduced weight less than did 1.8 mg liraglutide (WMD, 0.71 kg; 95% CI, 0.10 to 1.32^14^. Compared with glargine, dulaglutide lowered body weight significantly (WMD, −2.52kg; 95% CI, −3.35 to −1.70)^16^,^17^,^18^.

### Hypoglycemia

Hypoglycemia was defined as a plasma glucose ≤3.9 mmol/L (≤70 mg⁄ dL) with or without symptoms. Severe hypoglycemia was defined as an episode that required the assistance of another person to actively administer therapy^20^.

When administered as a monotherapy, the incidence of hypoglycemia with dulaglutide (*n* = 1059) and control (*n* = 544; placebo, metformin and liraglutide) was 7.8% and 10.6%, respectively; there was no difference between groups (RR, 1.07; 95% CI, 0.80 to 1.44) (see Supplementary Fig. S3 online)^9^,^12^,^15^,^19^. A total of 3.7% of patients in the dulaglutide group experienced hypoglycemia, compared with 1.4% in the placebo group; the incidence of hypoglycemia was higher in the dulaglutide-treated subjects (RR, 2.58; 95% CI, 1.05 to 6.31)^9^,^15^,^19^. In addition, the incidence of hypoglycemia was 11.7% and 12.7% with dulaglutide and metformin monotherapy, respectively; there was no significant difference between groups (RR, 0.92; 95% CI, 0.67 to 1.27)^12^. The incidence of hypoglycemia was 2.1% and 1.5% in dulaglutide and liraglutide group, no significant differences were noted (RR, 1.48; 95% CI, 0.29 to 7.42)^19^.

When used as an add-on therapy to OAM and lispro, dulaglutide led to the similar incidence of hypoglycemia notably compared with control (placebo, sitagliptin, exenatide, liraglutide and glargine) (24.5% vs. 24.5%, respectively; RR, 1.07; 95% CI, 0.89 to 1.30) (see Supplementary Fig. S4 online)^8^,^11^,^13^,^14^,^16^,^17^,^18^. Compared with placebo, dulaglutide as an add-on to OAM was associated with a higher incidence of hypoglycemia (13.4% vs. 6.9%, respectively; RR, 1.82; 95% CI, 1.44 to 2.31)^8^,^11^,^13^. When used as an add-on to metformin, the incidence of hypoglycemia with 0.75 mg dulaglutide was 5.3%, compared with 4.8% with 100 mg QD with sitagliptin; no difference was noted (RR, 1.11; 95% CI, 0.56 to 2.21). The incidence of hypoglycemia with 1.5 mg dulaglutide was 10.2%, which was higher than that observed with sitagliptin (RR, 2.14; 95% CI, 1.18 to 3.89)^11^. When used as an add-on to metformin, there was no significant difference in the occurrence of hypoglycemia in 26 (9%) patients given 1.5 mg dulaglutide and 17 (6%) patients given 1.8 mg liraglutide (RR, 1.59; 95% CI, 0.84 to 2.99). Compared with glargine, dulaglutide increased the incidence of hypoglycemia (WMD, 0.69; 95% CI, 0.56 to 0.85)^16^,^17^,^18^.

### Gastrointestinal disorders

Gastrointestinal disorders, such as nausea, vomiting, and diarrhea, were common in these trials. The percentage of patients who presented with nausea, vomiting, and diarrhea was 11.2%, 7.3%, and 5.8%, respectively in patients treated with dulaglutide monotherapy, compared with 10.9%, 2.6%, and 4.3% in the control group (placebo, metformin and liraglutide); no differences were noted between the two groups except the percentage of vomiting.

When used as an add-on therapy to OAM and lispro, dulaglutide increased the risk of nausea (17.3% vs. 9.0%; RR, 2.64; 95% CI, 1.69 to 4.12), vomiting (10.0% vs. 7.2%; RR, 2.58; 95% CI, 1.53 to 4.35), and diarrhea (12.0% vs. 5.9%; RR, 2.04; 95% CI, 1.57 to 2.65) significantly compared with control (placebo, sitagliptin, exenatide, liraglutide and glargine).

### Dose-effect relationships

The fixed dose scheme of 0.75 mg QW, 1.0 mg QW, and 1.5 mg QW dulaglutide was analyzed to assess for dose-effect relationships. Compared with control (placebo, metformin and liraglutide), 0.75 mg QW, 1.0 mg QW, and 1.5 mg QW dulaglutide monotherapy revealed decreases in HbA1c of 0.60%, 1.03%, and 0.56% (Fig. 3); reductions in FPG of 0.73 mmol/L, 1.45 mmol/L, and 0.98 mmol/L (Fig. 5), except the changes of bodyweight was 0.50 kg, 0.30 kg, and −0.08 kg (see Supplementary Fig. S1 online), respectively. Regarding hypoglycemia, there was no dose-effect relationship with the three doses of dulaglutide. Subgroup analyses indicated that the RR values for 0.75 mg QW, 1.0 mg QW, and 1.5 mg QW dulaglutide were 0.98, 2.67, and 1.03, respectively. When gastrointestinal disorders (nausea, vomiting) were considered there was no dose-effect relationship with 0.75 mg QW, 1.0 mg QW, and 1.5 mg QW dulaglutide. Subgroup analyses revealed that the respective RR values were 0.81, 1.25, and 1.20 for nausea, 1.84, 0.19, and 1.88 for vomiting.

Compared with control (placebo, sitagliptin, exenatide, liraglutide and glargine), as an add-on therapy to OAM and lispro, 0.75 mg QW, 1.0 mg QW, and 1.5 mg QW dulaglutide revealed reduction in HbA1c of 0.33%, 1.08%, and 0.43% (Fig. 4); except the changes of bodyweight was −1.16 kg, −1.22 kg, and −1.45 kg (see Supplementary Fig. S2 online), respectively. Regarding hypoglycemia, there was no dose-effect relationship with the three doses of dulaglutide. Subgroup analyses indicated that the RR values for 0.75 mg QW, 1.0 mg QW, and 1.5 mg QW dulaglutide were 0.82, 2.22, and 1.19, respectively. When gastrointestinal disorders (nausea, vomiting, and diarrhea) were considered there was no dose-effect relationship with 0.75 mg QW, 1.0 mg QW, and 1.5 mg QW dulaglutide. Subgroup analyses revealed that the respective RR values were 2.88, 2.23, and 2.88 for nausea, 2.59, 0.51, and 2.97 for vomiting, and 2.16, 0.81, and 2.19 for diarrhea.

### Pancreatitis

Seven cases of pancreatitis were reported in the included studies. Of these, two were considered to be related to the study drug^8^, two were in a sitagliptin arm^11^, one was in a placebo arm^9^, one in 1.5 mg dulaglutide arm, one in AWARD-2^16^, and one was in the placebo group during the sitagliptin period^11^. One patient who had no signs and symptoms of pancreatitis before the study was diagnosed with chronic pancreatitis ~7 months after treatment in the 1.5 mg dulaglutide arm^13^. No cases of adjudicated pancreatitis were reported during one study^12^, and no information was available from two trials^10^.

## Discussion

This study was a meta-analysis to assess the efficacy and safety of the GLP-1 receptor agonist dulaglutide, which was prescribed to subjects with type 2 diabetes with or without other hypoglycemic drugs.

GLP-1 receptor agonists showed a superior ability to lower HbA1c levels. A small number of meta-analyses showed that GLP-1 receptor agonists reduced HbA1c by ~1% compared with placebo^21^,^22^. Specifically, the proportion of patients achieving an HbA1c <7% was 46% for exenatide, 47% for liraglutide, and 63% for exenatide long-acting release^23^. Compared with control, as a monotherapy and as an add-on to OAM and lispro, dulaglutide reduced HbA1c significantly by −0.68% and −0.51%, respectively. As a monotherapy, dulaglutide resulted in an increased number of patients that achieved an HbA1c <7% (62.3% vs. 44.9%) and ≤6.5% (40.5% vs. 27.0%). As an add-on to OAM and lispro, dulaglutide also increased the number of patients that reached the HbA1c target of <7.0% (55.6% vs. 43.6%); a similar percentage of patients achieved and HbA1c ≤6.5% in both groups (36.9% vs. 32.1%). In head-to-head comparisons with exenatide (10 μg BID), 0.75 mg and 1.5 mg dulaglutide lowered HbA1c (pooled WMD = −0.41%) and increased the percentage of patients that achieved an HbA1c target <7% (64.8% vs. 48.9%). Dulaglutide (1.5 mg), as an add-on to metformin, was not inferior to once-daily1.8 mg liraglutide for reducing HbA1c levels (−1.42% vs. −1.36%, respectively). Compared with liraglutide (0.9 mg), dulaglutide (0.75 mg) monotherapy once weekly led to the similar reduction of HbA1c (−1.43% vs. −1.33%). As for sitagliptin, dulaglutide exhibited virtue in HbA1c control (the pooled WMD was −0.56%).

Bariatric surgery has been reported to ameliorate type 2 diabete with serious obesity. A retrospective trial evaluated the clinical efficacy of bariatric surgery vs liraglutide in patients with severely obese type 2 diabetic patients and found bariatric surgery lowered body weight and improved metabolic control than liraglutide significantly^24^. In the included studies, no evidences were found to compare dulaglutide with bariatric surgery directly. Compared with liraglutide, dulaglutide once weekly leads to the similar reduction of HbA1c, we speculate that bariatric surgery reduce HbA1c obviously than dulaglutide but it should be verified with clinical trials.

Hypoglycemia is a challenge and obstacle for the treatment of diabetes. When administered as a monotherapy, the risk of hypoglycemia was similar between the dulaglutide and control groups (placebo, metformin and liraglutide) (7.8% vs. 10.6%, respectively). When administered as an add-on therapy with OAM and lispro dulaglutide also did not increasethe risk of hypoglycemia (24.5% vs. 24.5%) compared with control (placebo, sitagliptin, exenatide, and liraglutide).

T2DM increases morbidity and mortality, mainly due to cardiovascular and cerebrovascular disease. The specific risk factors include weight gain, hypertension, and hyperlipidaemia. Therefore, antidiabetic drugs should be beneficial for cardiovascular and cerebrovascular diseases and also lower blood glucose. Obesity is associated with an increased risk for the development of hypertension, diabetes, dyslipidemia, and cardiovascular disease^25^. Weight gain is observed commonly in patients that use antidiabetic drugs, such as sulphonylureas, thiazolidinediones, glinides, and insulin; therefore, the correct drug should be selected carefully. GLP-1 receptor agonists (liraglutide, albiglutide, exenatide long-acting release, or exenatide^26^,^27^,^28^,^29^,^30^), have favorable weight profiles in patients with type 2 diabetes mellitus. When administered as a monotherapy, dulaglutide did not reduce weight compared with control (placebo, metformin and liraglutide). However, when dulaglutide is added to an OAM and lispro, it lowered bodyweight by 1.30 kg compared with control (placebo, sitagliptin, exenatide, and liraglutide and glargine).

Consistent with other GLP-1 receptor agonists^31^, the most common reported adverse events with dulaglutide were gastrointestinal disorders, particularly nausea, vomiting, and diarrhea. When used as a monotherapy, there were no differences in the rate of nausea (11.2% vs. 10.9%), and diarrhea (5.8% vs. 4.3%) except vomiting (7.3% vs. 2.6%) between the dulaglutide and control groups, respectively. However, when dulaglutide was added to OAM and lispro dulaglutide increased the risk of nausea (17.3% vs. 9.0%), vomiting (10.0% vs. 7.2%), and diarrhea (12.0% vs. 5.9%) obviously compared with control.

Of the included trials, three studies reported a significant dose-dependent reduction in HbA1c (dulaglutide 0.5 mg, 1.0 mg, and 1.5 mg^9^; dulaglutide 0.25 mg, 0.5 mg, and 0.75 mg^15^), FPG (dulaglutide 0.5 mg, 1.0 mg, and 1.5 mg^9^) and weight loss (LY2189265 0.5/1.0 mg, 1.0/1.0 mg, and 1.0/2.0 mg^8^). There were no dose-effect relationships with 0.75 mg QW, 1.0 mg QW, and 1.5 mg QW dulaglutide, whether administered as a monotherapy or as an add-on to another OAM and lispro, and control (placebo, metformin, sitagliptin, exenatide, liraglutide and glargine) regarding the reduction in HbA1c, FPG, the incidence of hypoglycemia, and the number of patients that experienced nausea and vomiting, except weight change. Therefore, additional RCTs are needed to clarify the dose-dependent effects of dulaglutide in the reduction in HbA1c and other parameters.

The risk of pancreatitis caused by incretin-based drugs used to treat diabetes is controversial, and the focus of international debates^32^,^33^. The FDA has made persistent efforts to estimate the risk of pancreatitis associated with incretin-based mimetic drugs. Current evidence does not support the view that incretin mimetic drugs are associated with an increased risk of pancreatitis^34^,^35^. In the current analysis the incidence of pancreatitis was rare in the dulaglutide arms; therefore, a causal relationship cannot be confirmed. And now, no evidences are found consuimg alcohol increases pancreatitis in the use of dulaglutide.

Up to date, safety and utility of dulaglutide were estimated in subjects with age more than 50 years, whether it shows the similar safety and utility in youth onset type 2 DM or people with age less than 50 years is unknown and needed to be evaluated in the future.

While most of the included studies on dulaglutide have been carried out on patients with HbA1c around 8–8.5%, there is some gap on the efficacy and safety. Mean duration of T2DM in the included studies was from about 3 to 12 years, which maybe one of the causes. With the extension of the duration, beta cell function decreases, this may lower dulaglutide efficacy because it reduces glucose through stimulation of islet cell secreting insulin. Second, doses of dulaglutide are various: 0.1 mg to 1.5 mg, that can lead to different efficacy and safety. Moreover, body weight, about 72 to 98 kg in our article, is associated with insulin sensitivity negatively, which can influence the efficacy of antidiabetic drug such as GLP-1 receptor agonists, insulin, metformin, and thiazolidinedione.

GLP-1 receptor agonists as the new agents for the treatment of T2DM, cost-benefit is an important topic. A retrospective cohort study estimated the cost effectiveness of treating patients to glycemic goal with Liraglutide versus Exenatide in a real-world clinical background and found that total diabetes related pharmacy costs per patient with the goal of HbA1c <7% were lower when Liraglutide was used than Exenatide ($3,108 vs. $3,354; P < 0.0001)^36^. Up to now, no data explore the cost consideration versus benefit of use of dulaglutide and further researches are needed to estimate this scope of dulaglutide.

### Limitations

Two of the included studies were Abstracts; therefore, some data could not be extracted and so some important information was lost. Moreover, only one trial provided data regarding renal function (serum creatinine <1.5 mg/dL [males] or <1.4 mg/dL [females]). Unlike endogenous GLP-1, dulaglutide is resistant to degradation by DPP-4; it is a large molecule with a delayed absorption rate and slower renal clearance. However, it remains unclear whether dulaglutide can be used in subjects with impaired renal function, and this should be evaluated. In addition, only a small number of articles reported fasting and postprandial blood glucose levels; therefore, we could not estimate postprandial changes after the administration of dulaglutide.

## Conclusions

This meta-analysis evaluated the efficacy and safety of dulaglutide for the treatment of type 2 diabetes. First, compared with control, monotherapy dulaglutide exhibited beneficial effects regarding the control of HbA1c, had a similar risk of hypoglycemia and gastrointestinal disorders, and a less body weight reduction. Furthermore, when used as an add-on therapy to OAM and lispro, dulaglutide lowered HbA1c and bodyweight, brought similar risk of hypoglycemia and gastrointestinal disorders compared with control. Moreover, there were no dose-dependent relationships with 0.75 mg QW, 1.0 mg QW, and 1.5 mg QW dulaglutide either as a monotherapy or as an add-on intervention regarding HbA1c, FPG, the incidence of hypoglycemia, and the number of patients that experienced nausea, vomiting except weight loss, compared with control. In addition, further particularly long-term studies are needed to fully appraise the benefit/risk profiles of dulaglutide, which will help determine the superiority of dulaglutide.

## Methods

### Outcomes measures of efficacy and safety

The primary outcome of efficacy was the change in HbA1c from baseline to the end of the trials. The secondary endpoints were the percentage of participants achieving HbA1c value <7% or ≤6.5%, fasting plasma glucose, and bodyweight. The safety and tolerability events were defined as any adverse events, symptomatic hypoglycemia, gastrointestinal disorders, and suspected pancreatitis.

### Eligibility criteria

All randomized clinical trials (RCTs) that lasted at least 12 weeks and analyzed dulaglutide as a monotherapy or as an add-on to other hypoglycemic drugs compared with placebo or other active drugs were included. The included subjects were nonpregnant adults with type 2 diabetes that had been diagnosed according to American Diabetes Association (1997) or World Health Organization criteria (1999). Reviews, letters, case reports, non-human studies, and trials that lasted less than 12 weeks were excluded. Our article is meta-analysis, which is not related to ethics.

### Search strategy

Medline (via PubMed), Embase (via OVID), the Cochrane Library and www.clinicaltrials.gov were searched until February 15^th^, 2015. The search results were limited to studies performed in humans, and did not restrict the language. The search terms used were “dulaglutide” and “LY2189265”, which were adjusted to comply with the relevant provisions in each database.

### Data extraction

Two reviewers performed data extraction independently according to the inclusion and exclusion criteria. The extracted data included study design, baseline characteristics, interventions, efficacy outcomes, safety, and tolerability. Any discrepancies between the two reviewers were resolved by consensus in the presence of a third reviewer when necessary.

### Risk of bias

The criteria used to assess the risk of bias of RCTs were from the Cochrane Collaboration’s risk of bias tool, and included random sequence generation, allocation concealment, blinding of outcome assessors, selective outcome reporting, and other items such as the funding source of the studies. Studies were graded as low, high, or unclear risk of bias. The two authors assessed the risk of bias and came to a consensus, and consulted a third reviewer to resolve any persistent disagreements.

### Data synthesis and analysis

Continuous data were analyzed using mean differences (MDs) to express effect size, and relative risk (RR) was used to express dichotomous data. An inconsistency index (I^2^), which is used to evaluate the heterogeneity of treatment effects, <25%, 25–50%, and >50% were considered as low, moderate, and high heterogeneity, respectively. A fixed-effects model was used for analysis if I^2^ <50%. In studies in which heterogeneity was identified, we searched for the sources of heterogeneity and subgroup analysis was considered or a random effects model was used. All analyses were performed using Review Manager V.5.2 statistical software.

## Additional Information

**How to cite this article**: Zhang, L. *et al.* Efficacy and safety of dulaglutide in patients with type 2 diabetes: a meta-analysis and systematic review. *Sci. Rep.* **6**, 18904; doi: 10.1038/srep18904 (2016).

## Supplementary Material

Supplementary Information

## Figures and Tables

**Table 1:** Baseline characteristics of the study population included in the meta-analysis.

Primary study	Study duration(weeks)	Source of information	Study arms included in meta-analyses	No. of patients randomised	HbA1c (%)	Mean years (years)	Mean duration of T2DM (years)	Weight (kg)	Background treatment
Umpierrez 2011	16 Phase 2	, NCT00630825	LY2189265 0.5/1.0 mg	66	8.05 ± 0.8	56 ± 12	7.5 ± 5.4	94.7 ± 15	Met + SU Met + TZD Met + DPP-IV inhibitors Other OAD
			LY2189265 1.0/1.0 mg	66	8.25 ± 0.9	59 ± 12	9.0 ± 7.6	94.8 ± 17	
			LY2189265 1.0/2.0 mg	65	8.25 ± 1.0	57 ± 12	8.1 ± 5.4	96.7 ± 17	
			placebo	66	8.43 ± 1.0	54 ± 11	8.6 ± 6.9	98.6 ± 18.4	
Grunberger 2012	12 Phase 2	NCT00791479	du 0.1 mg	35	7.4 ± 0.6	55.0 ± 9.3	3.9 ± 4.7	90.9 ± 18.9	medication-naı¨ve or had discontinued metformin monotherapy
			du 0.5 mg	34	7.1 ± 0.6	56.3 ± 9.2	3.9 ± 3.2	87.1 ± 17.3	
			du 1.0 mg	34	7.2 ± 0.6	56.9 ± 9.1	3.7 ± 3.8	90.2 ± 21.3	
			du 1.5 mg	29	7.3 ± 0.4	57.5 ± 7.9	4.6 ± 4.1	85.8 ± 18.6	
			placebo	32	7.3 ± 0.7	57.2 ± 8.8	3.3 ± 2.5	86.9 ± 17.0	
Wysham 2014 (AWARD-1)	52 Phase 3	NCT01064687	du 0.75 mg	280	8.1 ± 1.2	56 ± 9	9 ± 5	96 ± 21	Metformin + pioglitazone
			du 1.5 mg	279	8.1 ± 1.3	56 ± 10	9 ± 6	96 ± 20	
			Exenatide	276	8.1 ± 1.3	55 ± 10	9 ± 6	97 ± 19	
			placebo	141	8.1 ± 1.3	55 ± 10	9 ± 6	94 ± 19	
Guerci2013	104 phase 3	NCT00734474	du 0.75 mg	302	8.2 ± 1.1	54 ± 10	7 ± 5	86 ± 18	metformin
			du 1.5 mg	304	8.1 ± 1.1	54 ± 10	7 ± 6	87 ± 17	
			sitagliptin	315	8.1 ± 1.1	54 ± 10	7 ± 5	86 ± 17	
Nauck2014 (AWARD-5)	52 phase 3	NCT00734474	du 0.75 mg	302	8.2 ± 1.1	54 ± 10	7 ± 5	86 ± 18	metformin
			du 1.5 mg	304	8.1 ± 1.1	54 ± 10	7 ± 6	87 ± 17	
			sitagliptin	315	8.1 ± 1.1	54 ± 10	7 ± 5	86 ± 17	
			placebo	177	8.1 ± 1.1	55 ± 9	7 ± 5	87 ± 17	
Umpierrez 2014 (AWARD-3)	52 phase 3	NCT01126580	du 0.75 mg	270	7.6 ± 0.9	56 ± 11	3 ± 2	92 ± 19	diet and exercise
			du 1.5 mg	269	7.6 ± 0.9	56 ± 10	3 ± 2	93 ± 19	
			metformin	268	7.6 ± 0.8	55 ± 10	3 ± 2	92 ± 19	
Dungan 2014 (AWARD-6)	26 phase 3	NCT01624259	du 1.5 mg	299	8·1% ± 0·8	56·5 ± 9·3	7·1 ± 5·4	93.8 ± 18.2	metformin
			liraglutide 1·8 mg	300	8·1% ± 0·8	56·8 ± 9·9	7·3 ± 5·4	94·4 ± 19·0	
Terauchi 2014	12 phase 2	NCT01001104	du 0.25 mg	36	8.1 ± 0.7	52.3 ± 8.8	4.3 ± 3.5	74.0 ± 14.5	medication- naïve
			du 0.5 mg	37	8.0 ± 0.7	52.5 ± 9.2	4.9 ± 4.0	72.1 ± 12.8	
			du 0.75 mg	35	8.0 ± 0.6	52.2 ± 7.8	4.6 ± 4.5	75.8 ± 10.8	
			placebo	37	8.0 ± 0.6	51.7 ± 9.7	4.6 ± 4.1	76.4 ± 15.9	
AWARD-2	78 phase 3	NCT01075282	du 0.75 mg	272	8.13 ± 0.98	56.56 ± 9.27	9.28 ± 5.93	86.18 ± 18.15	Metformin Glimepiride
			du 1.5 mg	273	8.18 ± 1.03	56.24 ± 9.76	9.13 ± 6.22	85.13 ± 17.90	
			glargine	262	8.10 ± 0.95	57.21 ± 9.38	8.87 ± 5.98	87.66 ± 19.62	
AWARD-4	52 phase 3	NCT01191268	du 0.75 mg	293	8.40 ± 1.03	59.31 ± 8.98	12.43 ± 6.92	91.69 ± 18.03	Lispro
			du 1.5 mg	295	8.46 ± 1.08	58.88 ± 9.55	12.80 ± 7.19	91.00 ± 18.24	
			glargine	296	8.53 ± 1.03	59.90 ± 9.08	12.96 ± 6.80	90.75 ± 18.87	
	26 phase 3	NCT01584232	du 0.75 mg	181		57.52 ± 10.48			sulfonylureas and/or biguanides
			glargine	180		56.14 ± 11.33			
	26 phase 3	NCT01558271	du 0.75 mg	280		57.15 ± 9.57			
			liraglutide 0.9 mg	137		57.91 ± 10.93			medication- naïve
			Placebo	70		57.66 ± 8.34			

LY2189265/du = dulaglutide.
