# Supplementary material for: Efficacy and safety of dulaglutide in patients with type 2 diabetes: a meta-analysis and systematic review
Source: Sci Rep. 2016 Jan 8;6:18904. doi: 10.1038/srep18904 (PMC4705511; doi:10.1038/srep18904)
Supplement: Supplementary Information [file srep18904-s1.pdf]

Efficacy and safety of dulaglutide in patients with type 2 diabetes: a meta-analysis and systematic review

Lin Zhang[1+], Mei Zhang[2+], Yuwei Zhang[1+], Nanwei Tong[1*]

1. Department of Endocrinology and metabolism,

2. Department of Laboratory Medicine, West China Hospital, Sichuan University, Chengdu 610041, Sichuan Province, P.R China

[*]**Corresponding author:** Nanwei Tong

Department of Endocrinology and Metabolism, West China Hospital of Sichuan University, Chengdu 610041, Sichuan Province, P.R China

E-mail: buddyjun@hotmail.com

Tel: + 86 189 8060 1196

[+]These authors contributed equally to this manuscript.

Study or Subgroup	Dulaglutide Mean	Dulaglutide SD	Dulaglutide Total	Control Mean	Control SD	Control Total	Weight	Mean Difference IV, Fixed, 95% CI
**2.5.1 Dulaglutide0.1mg**								
Grunberger2012	-0.2	0.37	35	-1.4	2.83	32	5.9%	1.20 [0.21, 2.19]
**Subtotal (95% CI)**			**35**			**32**	**5.9%**	**1.20 [0.21, 2.19]**
Heterogeneity: Not applicable								
Test for overall effect: Z = 2.38 (P = 0.02)								
**2.5.2 Dulaglutide 0.25mg**								
Terauchi2014	0.4	1.77	35	-0.8	1.82	37	8.4%	1.20 [0.37, 2.03]
**Subtotal (95% CI)**			**35**			**37**	**8.4%**	**1.20 [0.37, 2.03]**
Heterogeneity: Not applicable								
Test for overall effect: Z = 2.84 (P = 0.005)								
**2.5.3 Dulaglutide 0.5mg**								
Grunberger2012	-0.3	2.33	34	-1.4	2.83	32	3.7%	1.10 [-0.15, 2.35]
Terauchi2014	-0.4	1.8	36	-0.8	1.82	37	8.4%	0.40 [-0.43, 1.23]
**Subtotal (95% CI)**			**70**			**69**	**12.1%**	**0.61 [-0.08, 1.31]**
Heterogeneity: Chi² = 0.83, df = 1 (P = 0.36); I² = 0%								
Test for overall effect: Z = 1.74 (P = 0.08)								
**2.5.4 Dulaglutide 0.75mg**								
NCT01558271	-0.02	2.34	280	-0.45	2.36	206	32.3%	0.43 [0.01, 0.85]
Terauchi2014	-0.6	1.8	36	-0.8	1.82	37	8.4%	0.20 [-0.63, 1.03]
Umpierrez2014	-1.36	3.94	270	-2.22	3.93	268	13.1%	0.86 [0.19, 1.53]
**Subtotal (95% CI)**			**586**			**511**	**53.8%**	**0.50 [0.17, 0.83]**
Heterogeneity: Chi² = 1.73, df = 2 (P = 0.42); I² = 0%								
Test for overall effect: Z = 2.98 (P = 0.003)								
**2.5.5 Dulaglutide 1.0mg**								
Grunberger2012	-1.1	2.33	34	-1.4	2.83	32	3.7%	0.30 [-0.95, 1.55]
**Subtotal (95% CI)**			**34**			**32**	**3.7%**	**0.30 [-0.95, 1.55]**
Heterogeneity: Not applicable								
Test for overall effect: Z = 0.47 (P = 0.64)								
**2.5.6 Dulaglutide 1.5mg**								
Grunberger2012	-1.5	2.69	29	-1.4	2.83	32	3.0%	-0.10 [-1.49, 1.29]
Umpierrez2014	-2.29	3.94	269	-2.22	3.93	268	13.1%	-0.07 [-0.74, 0.60]
**Subtotal (95% CI)**			**298**			**300**	**16.1%**	**-0.08 [-0.68, 0.52]**
Heterogeneity: Chi² = 0.00, df = 1 (P = 0.97); I² = 0%								
Test for overall effect: Z = 0.25 (P = 0.80)								
**Total (95% CI)**			**1058**			**981**	**100.0%**	**0.51 [0.27, 0.75]**
Heterogeneity: Chi² = 10.95, df = 9 (P = 0.28); I² = 18%								
Test for overall effect: Z = 4.18 (P < 0.0001)								
Test for subgroup differences: Chi² = 8.39, df = 5 (P = 0.14), I² = 40.4%								

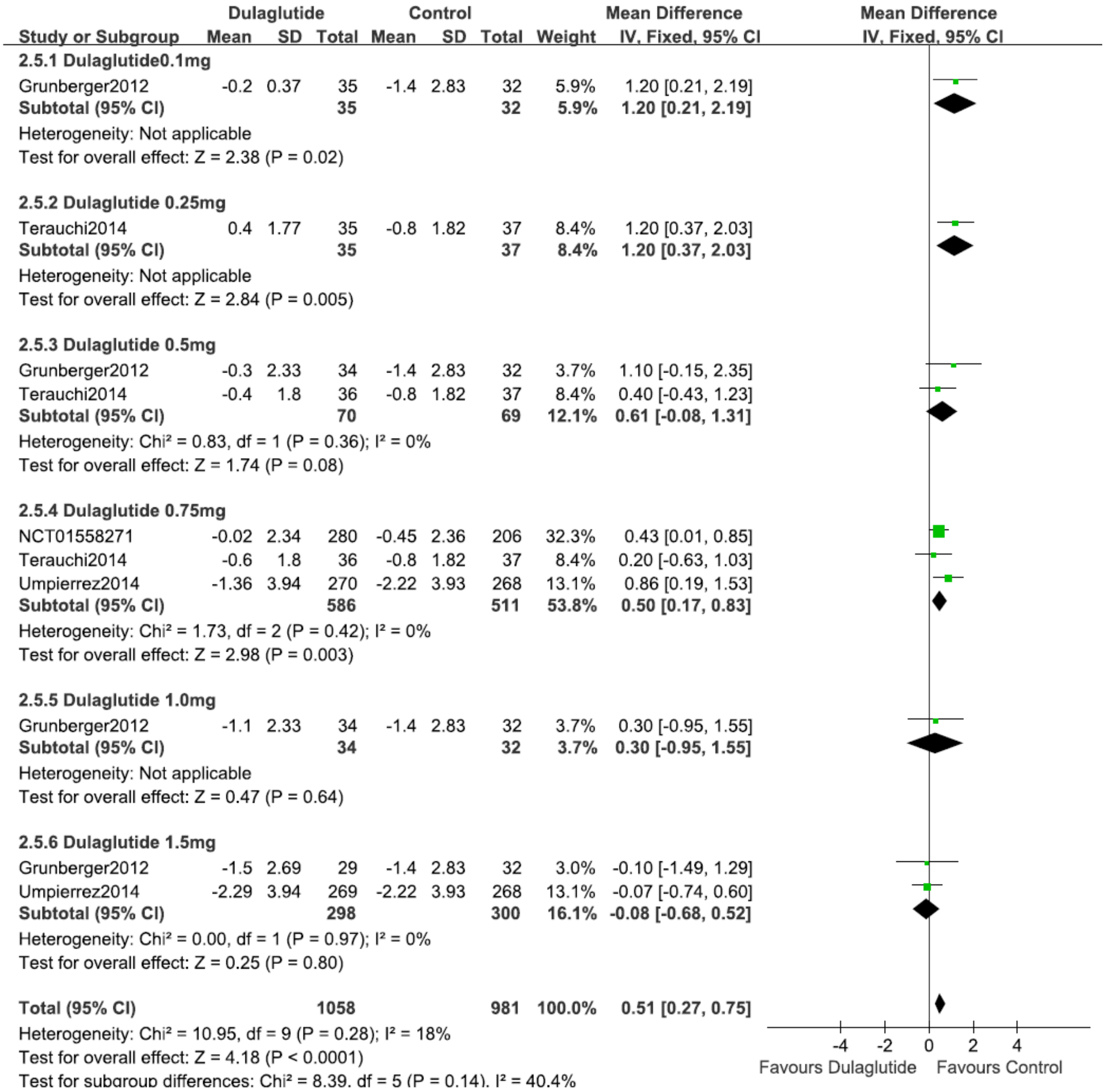

FigureS1. Bodyweight: dulaglutide monotherapy vs. control

Study or Subgroup	Dulaglutide Mean	Dulaglutide SD	Dulaglutide Total	Control Mean	Control SD	Control Total	Weight	Mean Difference IV, Random, 95% CI
**6.5.1 Dulaglutide0.75mg**								
Guerci2013	-2.39	4.52	302	-1.75	4.44	315	6.9%	-0.64 [-1.35, 0.07]
Nauck2014	-2.6	3.99	302	-1.53	3.9	315	7.0%	-1.07 [-1.69, -0.45]
NCT01075282	-1.54	4.27	270	1.28	4.18	259	6.8%	-2.82 [-3.54, -2.10]
NCT01191268	0.86	5.61	289	2.89	5.66	294	6.4%	-2.03 [-2.94, -1.12]
NCT01584232	-0.48	2.28	180	0.94	2.28	180	7.3%	-1.42 [-1.89, -0.95]
Wysham2014	0.54	6.34	280	-0.71	6.31	276	6.0%	1.25 [0.20, 2.30]
**Subtotal (95% CI)**			**1623**			**1639**	**40.4%**	**-1.16 [-2.03, -0.29]**
Heterogeneity: Tau² = 1.04; Chi² = 46.27, df = 5 (P < 0.00001); I² = 89%								
Test for overall effect: Z = 2.60 (P = 0.009)								
**6.5.2 Dulaglutide1.0mg**								
Umpierrez2011	-1.34	3.09	63	-0.12	3.14	65	6.0%	-1.22 [-2.30, -0.14]
**Subtotal (95% CI)**			**63**			**65**	**6.0%**	**-1.22 [-2.30, -0.14]**
Heterogeneity: Not applicable								
Test for overall effect: Z = 2.22 (P = 0.03)								
**6.5.3 Dulaglutide1.5mg**								
Dungan2014	-2.9	3.8	299	-3.61	3.81	300	7.1%	0.71 [0.10, 1.32]
Guerci2013	-2.88	4.36	304	-1.75	4.44	315	6.9%	-1.13 [-1.82, -0.44]
Nauck2014	-3.03	3.83	304	-1.53	3.9	315	7.1%	-1.50 [-2.11, -0.89]
NCT01075282	-1.96	4.29	272	1.28	4.18	259	6.8%	-3.24 [-3.96, -2.52]
NCT01191268	-0.35	5.75	286	2.89	5.66	294	6.3%	-3.24 [-4.17, -2.31]
Wysham2014	-1.01	6.35	279	-0.71	6.31	276	6.0%	-0.30 [-1.35, 0.75]
**Subtotal (95% CI)**			**1744**			**1759**	**40.2%**	**-1.45 [-2.72, -0.17]**
Heterogeneity: Tau² = 2.39; Chi² = 90.50, df = 5 (P < 0.00001); I² = 94%								
Test for overall effect: Z = 2.22 (P = 0.03)								
**6.5.4 Dulaglutide0.5/1.0mg**								
Umpierrez2011	-1.44	3.14	65	-0.12	3.14	65	6.0%	-1.32 [-2.40, -0.24]
**Subtotal (95% CI)**			**65**			**65**	**6.0%**	**-1.32 [-2.40, -0.24]**
Heterogeneity: Not applicable								
Test for overall effect: Z = 2.40 (P = 0.02)								
**6.5.5 Dulaglutide1.0/2.0mg**								
Umpierrez2011	-1.59	0.94	62	-0.24	0.97	65	7.5%	-1.35 [-1.68, -1.02]
**Subtotal (95% CI)**			**62**			**65**	**7.5%**	**-1.35 [-1.68, -1.02]**
Heterogeneity: Not applicable								
Test for overall effect: Z = 7.97 (P < 0.00001)								
**Total (95% CI)**			**3557**			**3593**	**100.0%**	**-1.30 [-1.85, -0.75]**
Heterogeneity: Tau² = 1.02; Chi² = 136.87, df = 14 (P < 0.00001); I² = 90%								
Test for overall effect: Z = 4.62 (P < 0.00001)								
Test for subgroup differences: Chi² = 0.23, df = 4 (P = 0.99), I² = 0%								

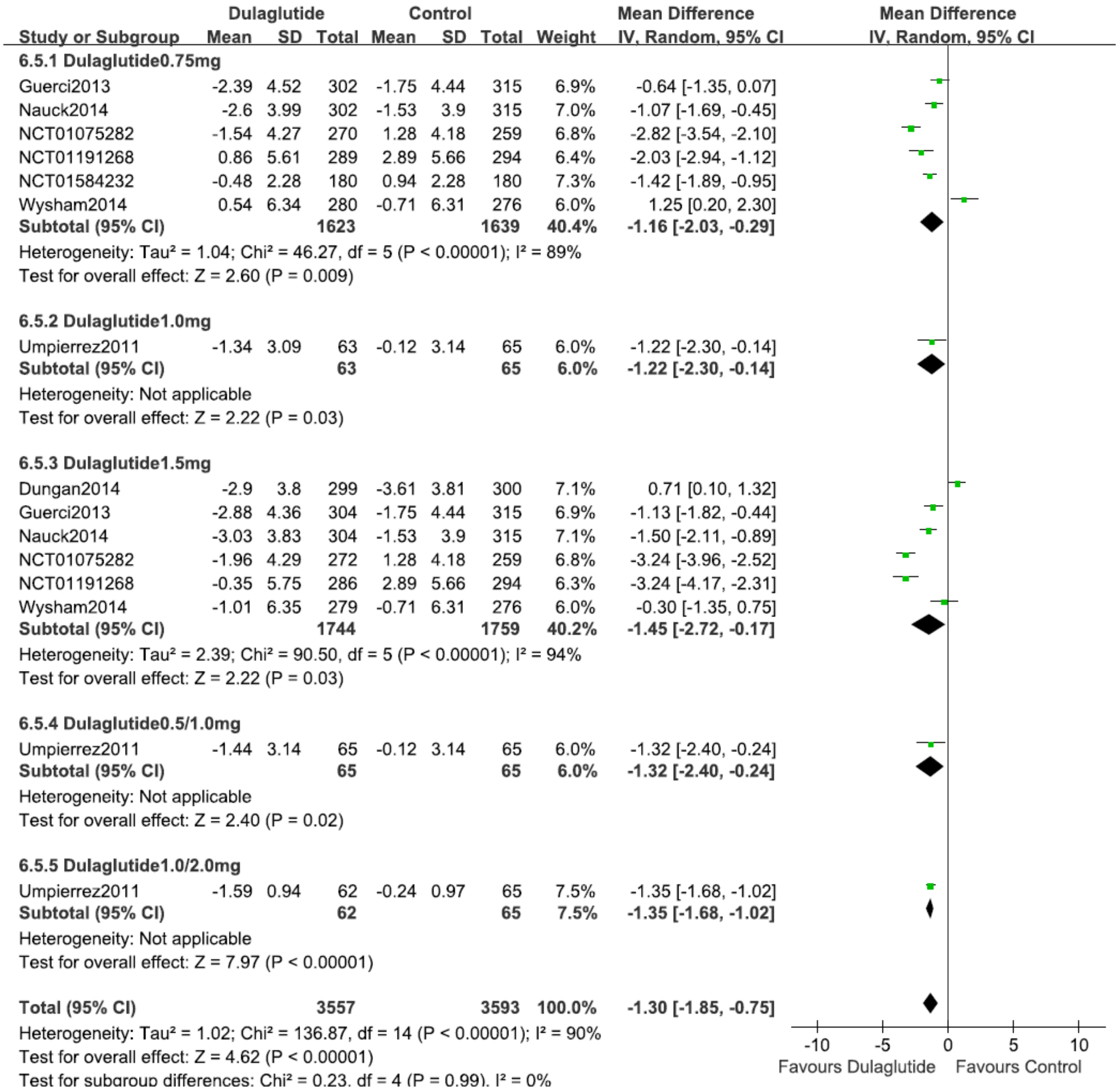

Figure S2. Body weight: dulaglutide add-on to active drugs vs. control

Study or Subgroup	Dulaglutide Events	Dulaglutide Total	Control Events	Control Total	Weight	Risk Ratio M-H, Fixed, 95% CI
**2.6.1 Dulaglutide 0.1mg**						
Grunberger2012	2	35	1	32	1.4%	1.83 [0.17, 19.21]
**Subtotal (95% CI)**		**35**		**32**	**1.4%**	**1.83 [0.17, 19.21]**
Total events	2		1			
Heterogeneity: Not applicable						
Test for overall effect: Z = 0.50 (P = 0.62)						
**2.6.2 Dulaglutide 0.25mg**						
Terauchi2014	0	35	0	37		Not estimable
**Subtotal (95% CI)**		**35**		**37**		**Not estimable**
Total events	0		0			
Heterogeneity: Not applicable						
Test for overall effect: Not applicable						
**2.6.3 Dulaglutide 0.5mg**						
Grunberger2012	3	34	1	32	1.3%	2.82 [0.31, 25.77]
Terauchi2014	1	37	0	37	0.7%	3.00 [0.13, 71.34]
**Subtotal (95% CI)**		**71**		**69**	**2.0%**	**2.88 [0.47, 17.66]**
Total events	4		1			
Heterogeneity: Chi² = 0.00, df = 1 (P = 0.98); I² = 0%						
Test for overall effect: Z = 1.14 (P = 0.25)						
**2.6.4 Dulaglutide 0.75mg**						
NCT01558271	6	280	3	204	4.5%	1.46 [0.37, 5.76]
Terauchi2014	2	36	0	37	0.6%	5.14 [0.26, 103.39]
Umpierrez2014	30	270	34	268	44.6%	0.88 [0.55, 1.39]
**Subtotal (95% CI)**		**586**		**509**	**49.8%**	**0.98 [0.64, 1.51]**
Total events	38		37			
Heterogeneity: Chi² = 1.72, df = 2 (P = 0.42); I² = 0%						
Test for overall effect: Z = 0.07 (P = 0.94)						
**2.6.5 Dulaglutide 1.0mg**						
Grunberger2012	2	24	1	32	1.1%	2.67 [0.26, 27.72]
**Subtotal (95% CI)**		**24**		**32**	**1.1%**	**2.67 [0.26, 27.72]**
Total events	2		1			
Heterogeneity: Not applicable						
Test for overall effect: Z = 0.82 (P = 0.41)						
**2.6.6 Dulaglutide 1.5mg**						
Grunberger2012	3	29	1	32	1.2%	3.31 [0.36, 30.08]
Umpierrez2014	33	269	34	268	44.5%	0.97 [0.62, 1.51]
**Subtotal (95% CI)**		**298**		**300**	**45.7%**	**1.03 [0.67, 1.59]**
Total events	36		35			
Heterogeneity: Chi² = 1.15, df = 1 (P = 0.28); I² = 13%						
Test for overall effect: Z = 0.14 (P = 0.89)						
**Total (95% CI)**		**1049**		**979**	**100.0%**	**1.07 [0.80, 1.44]**
Total events	82		75			
Heterogeneity: Chi² = 5.11, df = 8 (P = 0.75); I² = 0%						
Test for overall effect: Z = 0.47 (P = 0.64)						
Test for subgroup differences: Chi² = 2.10, df = 4 (P = 0.72), I² = 0%						

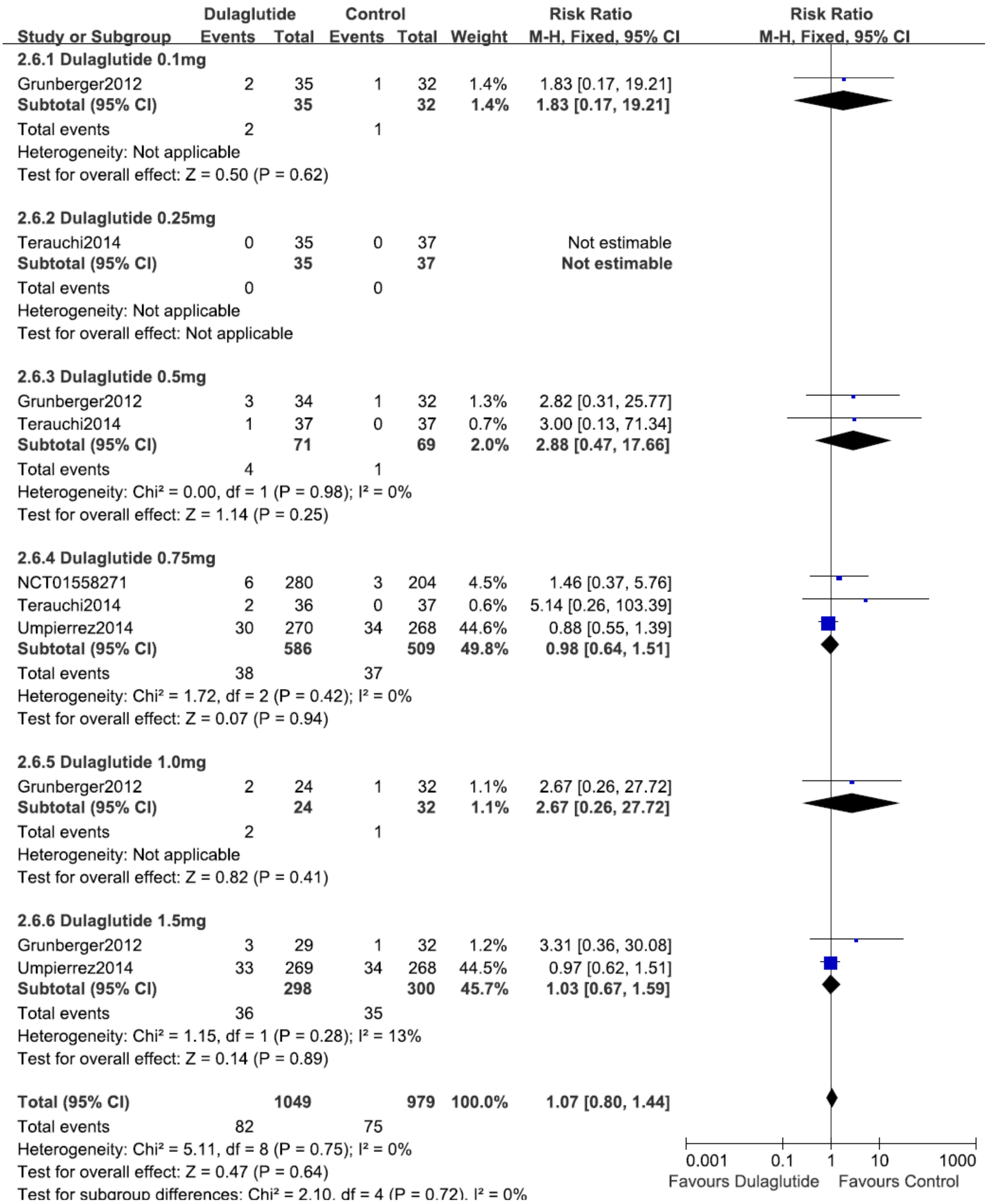

Figure S3. Hypoglycaemia: dulaglutide monotherapy vs. control

Study or Subgroup	Dulaglutide Events	Dulaglutide Total	Control Events	Control Total	Weight	Risk Ratio M-H, Random, 95% CI
**6.6.1 Dulaglutide0.75mg**						
Nauck2014	16	302	15	315	5.2%	1.11 [0.56, 2.21]
NCT01191268	196	293	207	296	15.6%	0.96 [0.86, 1.07]
NCT01584232	47	181	86	180	11.9%	0.54 [0.41, 0.73]
Wysham2014	30	280	49	417	8.9%	0.91 [0.59, 1.40]
**Subtotal (95% CI)**		**1056**		**1208**	**41.5%**	**0.82 [0.59, 1.16]**
Total events	289		357			
Heterogeneity: Tau² = 0.09; Chi² = 14.06, df = 3 (P = 0.003); I² = 79%						
Test for overall effect: Z = 1.11 (P = 0.27)						
**6.6.2 Dulaglutide1.0mg**						
Umpierrez2011	35	65	16	66	7.9%	2.22 [1.37, 3.60]
**Subtotal (95% CI)**		**65**		**66**	**7.9%**	**2.22 [1.37, 3.60]**
Total events	35		16			
Heterogeneity: Not applicable						
Test for overall effect: Z = 3.24 (P = 0.001)						
**6.6.3 Dulaglutide1.5mg**						
Dungan2014	26	299	17	300	6.3%	1.53 [0.85, 2.77]
Nauck2014	31	304	15	315	6.2%	2.14 [1.18, 3.89]
NCT01191268	191	295	207	296	15.5%	0.93 [0.83, 1.04]
Wysham2014	29	279	49	417	8.8%	0.88 [0.57, 1.36]
**Subtotal (95% CI)**		**1177**		**1328**	**36.9%**	**1.19 [0.81, 1.75]**
Total events	277		288			
Heterogeneity: Tau² = 0.10; Chi² = 11.12, df = 3 (P = 0.01); I² = 73%						
Test for overall effect: Z = 0.89 (P = 0.37)						
**6.6.4 Dulaglutide0.5/1.0mg**						
Umpierrez2011	19	66	16	66	6.6%	1.19 [0.67, 2.10]
**Subtotal (95% CI)**		**66**		**66**	**6.6%**	**1.19 [0.67, 2.10]**
Total events	19		16			
Heterogeneity: Not applicable						
Test for overall effect: Z = 0.59 (P = 0.56)						
**6.6.5 Dulaglutide1.0/2.0mg**						
Umpierrez2011	23	65	16	66	7.0%	1.46 [0.85, 2.50]
**Subtotal (95% CI)**		**65**		**66**	**7.0%**	**1.46 [0.85, 2.50]**
Total events	23		16			
Heterogeneity: Not applicable						
Test for overall effect: Z = 1.38 (P = 0.17)						
**Total (95% CI)**		**2429**		**2734**	**100.0%**	**1.07 [0.89, 1.30]**
Total events	643		693			
Heterogeneity: Tau² = 0.06; Chi² = 39.91, df = 10 (P < 0.0001); I² = 75%						
Test for overall effect: Z = 0.74 (P = 0.46)						
Test for subgroup differences: Chi² = 11.40, df = 4 (P = 0.02), I² = 64.9%						

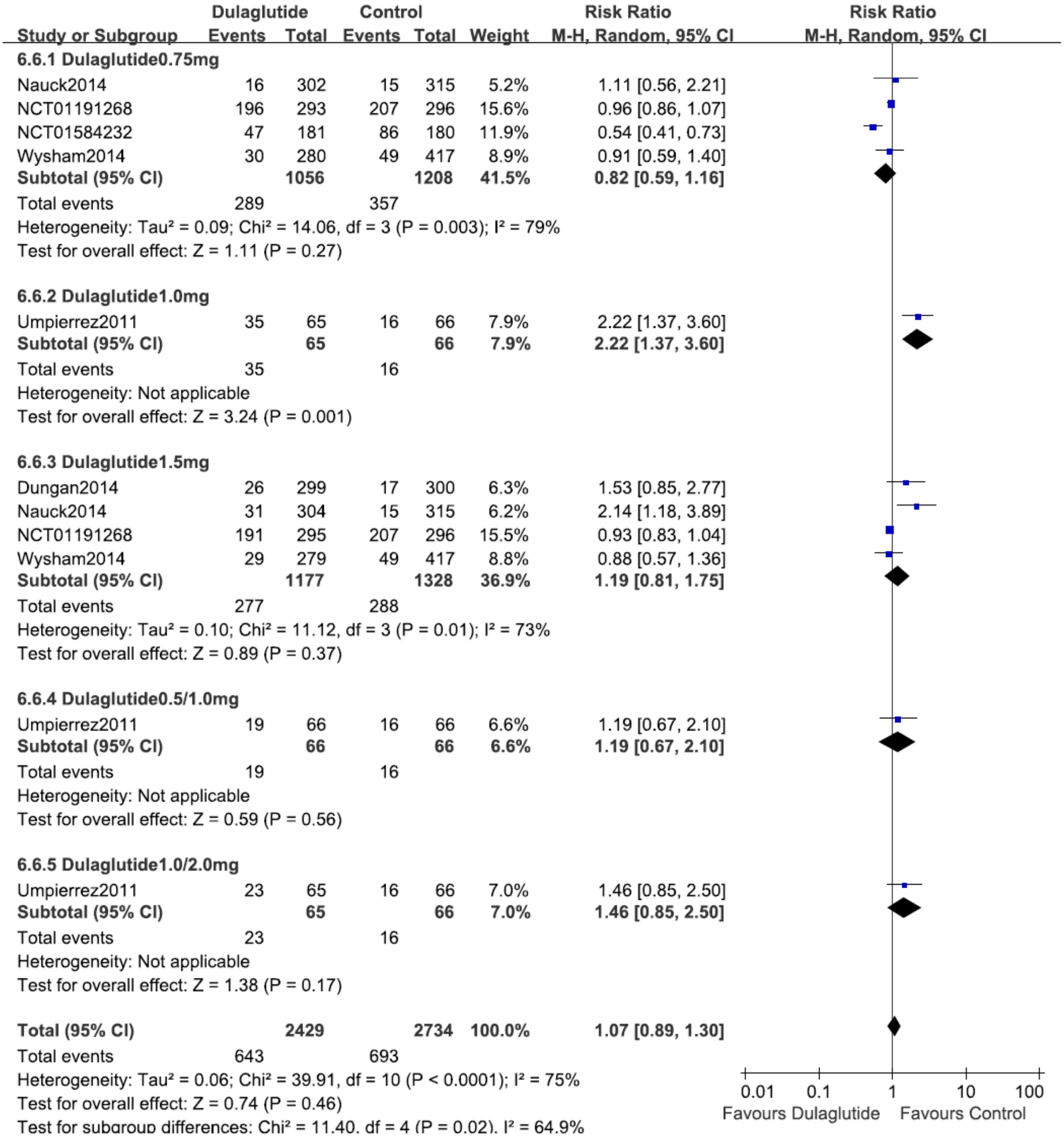

Figure S4. Hypoglycaemia: dulaglutide add-on to active drugs vs. control
